# Eco-friendly biosynthesis of silver nanoparticles by saprophytic *Fusarium solani* as a sustainable therapeutic approach for the management of methicillin-resistant *Staphylococcus aureus* wound infections

**DOI:** 10.1186/s12934-026-03126-3

**Published:** 2026-10-17

**Authors:** Heba A. Ramadan, Fatma Sonbol, Tarek El‑banna, Ahmed Kassem El-Sayed, Mohamed M. El-Zahed, Engy Elekhnawy

**Affiliations:** 1 Microbiology and Immunology Department, Faculty of Pharmacy, Tanta University, Tanta, 31527 Egypt; 2 Department of Clinical Research, Damietta Directorate of Health Affairs, Damietta, Egypt; 3 Department of Botany and Microbiology, Faculty of Science, Damietta University, New Damietta, 34517 Egypt; 4 Department of Basic Sciences, Faculty of Applied Health Sciences, Horus University-Egypt, New Damietta, 34517 Egypt; 5 Microbiology and Immunology Department, Faculty of Pharmacy, Alsalam University, Tanta, 31511 Egypt

**Keywords:** Biosynthesis, Silver nanoparticles, *Fusarium solani*, MRSA, Antibacterial activity, Wound healing

## Abstract

The rising prevalence of antimicrobial resistance (AMR) has prompted the exploration of novel, naturally sourced therapeutic alternatives. Here, the saprophytic fungus *Fusarium solani* was used to biosynthesize silver nanoparticles (AgNPs), which were characterized by UV–visible spectroscopy, transmission electron microscopy (TEM), scanning electron microscopy (SEM), X-ray diffraction (XRD), and Fourier-transform infrared spectroscopy (FTIR). The AgNPs showed a surface plasmon resonance peak at ~ 410 nm, a zeta potential of − 42.7 mV, an average TEM diameter of 19.58 nm, and a hydrodynamic (DLS) diameter of 121.4 nm with a polydispersity index of 0.302. Against clinical methicillin-resistant *Staphylococcus aureus* (MRSA) isolates, the AgNPs exhibited minimum inhibitory concentration (MIC) and minimum bactericidal concentration (MBC) values of 0.1875–0.375 mM, with confocal laser scanning microscopy (CLSM) showing a reduction in bacterial viability from 92.3% to 28.0% and TEM revealing marked ultrastructural membrane damage following treatment. In a murine MRSA wound infection model, AgNPs treatment increased wound healing from 12.33% to 95.33% and reduced bacterial load from 9.90 to 1.10 log CFU/g tissue relative to the untreated infected control, alongside significant downregulation of TNF-α, IL-6, and COX-2 expression and improved histopathological indices of tissue repair (all *p* < 0.05). These findings indicate that fungal-derived AgNPs possess potent antibacterial and wound-healing properties against MRSA, supporting their potential as a sustainable therapeutic candidate, although further mechanistic and translational studies are needed.

**Graphical abstract:**

**Figure:**
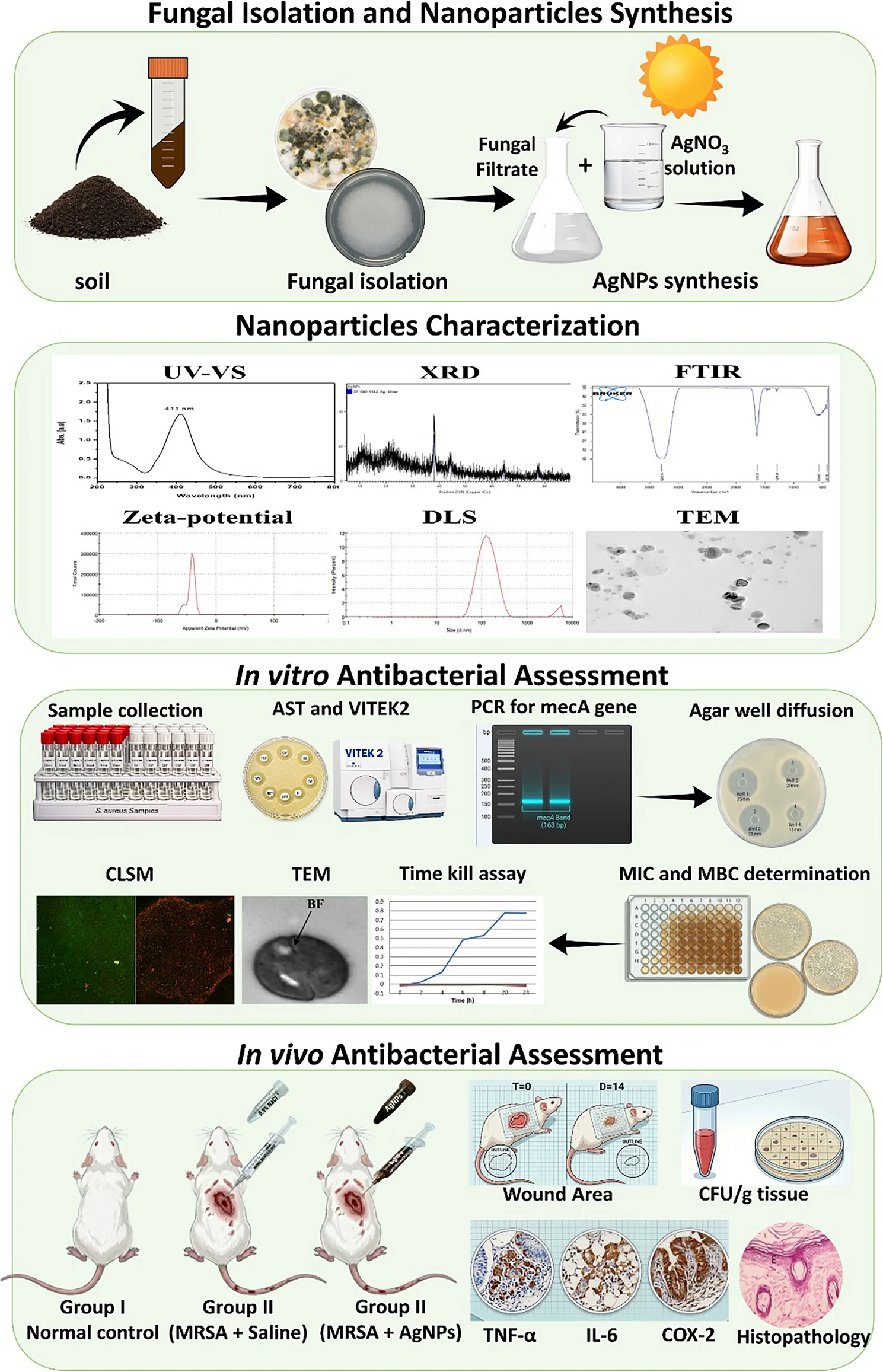

**Supplementary Information:**

The online version contains supplementary material available at https://doi.org/10.1186/s12934-026-03126-3.

## Introduction

Antimicrobial resistance (AMR) has become an emergent global health problem that threatens the utility of available antibiotics, leading to increased morbidity, increased mortality, and increased health care cost from bacterial infections [1, 2]. Among multidrug-resistant bacterial pathogens, methicillin-resistant *Staphylococcus aureus* (MRSA) remains one of the most clinically relevant threats. MRSA has also caused a broad variety of serious infections such as skin and soft tissue infections, pneumonia, bacteremia and endocarditis [3, 4]. MRSA is responsible for a substantial proportion of both hospital- and community-acquired infections worldwide, and its capacity for horizontal transfer of resistance determinants continues to complicate empirical treatment selection [3]. This clinical significance is strengthened by a rapid emergence of resistance, along with tissue persistence. Conventional antibiotic therapies against MRSA are increasingly compromised by reduced efficacy and dose-limiting toxicity [4].

All such challenges emphasize the crucial necessity for substitute antimicrobial approaches with mechanisms different from conventional antibiotics [5, 6]. Nanomaterials have attracted great consideration as promising antimicrobial agent owing to their broad spectrum of activity and multitargeting mechanisms of action against pathogens.

AgNPs are one of the metal NPs that have the ability to inhibit bacterial growth by employing multiple modes, including disturbance of bacterial membrane integrity, production of reactive oxygen species (ROS), interference with vital biomolecules, and inhibition of biofilm formation. The multitargeted modes of activity significantly lessen the rapid development of resistance against AgNPs [5, 7, 8].

However, the applications of AgNPs in biomedicine are often restricted by typical chemical and physical methods of synthesis. Such methods of NPs synthesis often employ toxic reagents, consume a high level of energy, and produce various environmentally hazardous products. Because of these disadvantages, the scientific community’s attention was drawn towards green synthesis, which provides a sustainable, low-cost, and eco-friendly technology. This approach has been extended beyond silver-based systems to other metal-oxide nanomaterials, such as zinc oxide nanoparticles, which have similarly demonstrated concentration-dependent antibacterial efficacy against diverse bacterial pathogens [6].

Fungi, in particular, have proved to have potential and excellent biological factories to synthesize NPs due to their capability of secreting great amounts of reducing enzymes, secondary metabolites, and proteins that behave as reducing and capping agents [9, 10].

Extracellular biosynthesis of AgNPs by fungi has several advantages, such as better NPs stability, controlled morphology, enhanced biocompatibility, and a lesser environmental impact. Although several fungal genera have been investigated for nanoparticle biosynthesis, *Fusarium solani* has received comparatively limited attention despite its demonstrated potential as a fungal biofactory for nanoparticle production [11]. Additionally, the research of locally isolated saprophytic fungi from agricultural soils is an unexplored source of new biosynthetic abilities and antimicrobial potential within the regional biodiversity [9, 12].

Despite these advantages, the biosynthesis of AgNPs by saprophytic fungi such as *Fusarium solani* and their antibacterial activity against MRSA infections have not been sufficiently illuminated, which is an important gap in current antimicrobial nanotechnology research, including in vitro and in vivo mechanistic insights [13]. Biosynthesized AgNPs derived from other microbial sources have also demonstrated promising antibacterial activity against multidrug-resistant bacteria isolated from chronic wounds [14]. However, comparable evidence for *Fusarium solani*-mediated AgNPs, particularly against MRSA and in conjunction with in vivo wound-healing evaluation, remains limited.

This study therefore aimed to isolate a saprophytic fungus from local agricultural soil, biosynthesize AgNPs, and evaluate their antibacterial efficacy against clinical MRSA isolates in vitro and in vivo, thereby assessing the potential of fungal-based green nanotechnology as a sustainable approach to MRSA-associated infections.

## Materials and methods

### Soil sampling and fungal isolation

Samples were collected at 5–10 cm depth in rhizosphere within a semi-natural habitat with introduced poultry, mature trees and nearby houses. Location of sampling site; Damietta, Egypt during August, 2024. The soil looked dark, moist and rich in decomposed organic matter, probably from accumulated animal waste and decomposing vegetation. Although no formal physicochemical analysis has been performed, the properties observed are suggestive of a biologically active substrate. The rhizosphere soil samples were collected with sterilized spatula and transferred into sterile polyethylene bags. In laboratory, 1 g of soil sample was mixed with 9 mL sterile distilled water and serially diluted till 10 − 4. Appropriate dilutions were plated in aliquots of 0.1 mL onto potato dextrose agar (PDA) supplemented with chloramphenicol (50 µg/mL) to inhibit bacterial growth. The plates were then incubated at 30 °C for 5–7 days and the developing fungal colonies were selected for further purification. Pure fungal colonies were subcultured and kept in PDA slants [15].

### Molecular identification of the fungal isolate

Genomic DNA was extracted from fungal mycelia using the DNeasy Plant Mini Kit (Qiagen, Germany). The internal transcribed spacer (ITS) region was amplified using universal primers ITS1 (5’-TCCGTAGGTGAACCTGCGG-3’) and ITS4 (5’-TCCTCCGCTTATTGATATGC-3’). PCR amplification was performed in a 25 µL total reaction volume using Emerald Amp GT PCR Master Mix (Takara, Japan), with thermal cycling conditions of initial denaturation at 94 °C for 5 min; 35 cycles of denaturation at 94 °C for 30 s, annealing at 56 °C for 40 s, and extension at 72 °C for 50 s; followed by a final extension at 72 °C for 10 min. PCR products were analyzed by electrophoresis on a 1.5% agarose gel stained with ethidium bromide and visualized under UV illumination, then purified and sequenced using the same primers [16]. Sequence analysis was performed using BLAST against the GenBank database for species identification.

### GC–MS analysis

The fungal isolate was cultured for extraction of secondary metabolites. Three fungal discs were inoculated into 100 mL of sterile broth medium and incubated on a rotary shaker at 28 ± 2 °C for 5–7 days for active metabolite production. Fungal biomass (pellets) was collected by filtration through sterile Whatman No. 1 filter paper and washed several times with sterile distilled water to remove media residues. The clean biomass was suspended in 30% methanol for 24 h to facilitate metabolite extraction. The mixture was then filtered, and the resulting filtrate was dried under dark conditions at room temperature to remove the solvent. The dried extract was re-dissolved in HPLC-grade methanol and analyzed by GC–MS to identify bioactive compounds potentially responsible for the reduction of silver ions during AgNPs biosynthesis.

### Green synthesis of AgNPs

The green synthesis of AgNPs was performed according to previously described methods [17, 18], with some modifications. In brief, the identified fungal isolate was grown in malt extract, yeast extract, glucose, and peptone (MYGP) broth and incubated for 5 days at 30 °C in a shaking incubator. The biomass was harvested, rinsed, and incubated in sterile distilled water for 24 h at 30 °C in a shaker. The supernatant was obtained by filtration using Whatman filter paper No. 1. The filtrate was mixed with an equal volume of 3 mM aqueous silver nitrate (AgNO₃) in a 250 mL Erlenmeyer flask at the native pH of the fungal filtrate without exogenous pH adjustment. The reaction mixture was exposed to direct sunlight for one hour. A visible colour change from pale yellow to dark brown was observed, indicating the formation of AgNPs. Sunlight was employed as a natural and sustainable energy source to accelerate the reduction of Ag⁺ ions, enhancing the efficiency and speed of NP synthesis [19, 20]. Control flasks lacking either the fungal filtrate or silver nitrate were maintained under identical conditions and did not exhibit any colour change.

### Characterization of AgNPs

#### Optical properties

UV–Vis absorption spectra were recorded using an Ocean Optics USB2000 + VIS-NIR fiber optic spectrophotometer in the range of 200–800 nm to confirm NPs formation via surface plasmon resonance (SPR).

#### X-ray diffraction (XRD) analysis

The crystalline structure of the synthesized AgNPs was analyzed using an XPERT-PRO powder X-ray diffractometer (PANalytical, Netherlands) with Cu Kα radiation (λ = 1.5406 Å). The diffraction patterns were recorded in the 2θ range of 20°–80° at a step size of 0.001°.

#### Fourier-transform infrared (FTIR) analysis

The FTIR spectra were recorded using a Bruker Vertex 70 (Bruker, Germany) in the range of 4000–400 cm⁻¹ to identify functional groups involved in the reduction and stabilization of AgNPs.

#### Hydrodynamic size and surface charge

Dynamic light scattering (DLS) and zeta potential measurements were performed using a Malvern Zetasizer Nano ZS (Malvern Instruments, UK). The sample homogeneity was evaluated using the polydispersity index (PDI).

#### Transmission electron microscopy (TEM)

TEM was performed on a Talos F200i high-resolution TEM (Thermo Fisher Scientific) at 200 kV. Samples were prepared by placing a drop of colloidal suspension onto Formvar carbon-coated 300-mesh copper grids (Ted Pella) and allowing them to dry at ambient temperature. Particle size distribution and average diameter were determined using image analysis software.

#### Elemental analysis

The elemental composition of the synthesized AgNPs was confirmed using energy-dispersive X-ray spectroscopy (EDS) attached to a scanning electron microscope (SEM) under appropriate accelerating voltage conditions.

### In vitro antibacterial activity

#### Collection and identification of bacterial isolates

*Staphylococcus aureus* isolates were recovered from clinical specimens collected from private outpatient clinics in Egypt. Specimen collection and identification are revealed in the supplementary file.

#### Antimicrobial susceptibility

Methicillin resistance was initially determined phenotypically and subsequently confirmed at the molecular level by PCR targeting the *mecA* gene (supplementary file).

Antimicrobial susceptibility profiling was evaluated using the Kirby-Bauer disk diffusion method in accordance with the guidelines of the Clinical and Laboratory Standards Institute (CLSI) [21]. The antimicrobial agents used were penicillin (PEN), oxacillin (OXA), gentamicin (GEN), ciprofloxacin (CIP), levofloxacin (LEV), moxifloxacin (MOX), erythromycin (ERY), clindamycin (CLI), quinupristin/dalfopristin (Q/D), linezolid (LNZ), vancomycin (VAN), tetracycline (TET), tigecycline (TGC), rifampicin (RIF), and trimethoprim/sulfamethoxazole (SXT). The plates were incubated at 37 °C for 18 h, and the diameters of the inhibition zones were measured to determine the bacterial susceptibility according to CLSI interpretive criteria.

#### Antibacterial action of AgNPs

Antibacterial activity of AgNPs was assessed utilizing the agar well diffusion technique. Mueller-Hinton agar plates were inoculated with bacterial suspensions calibrated at 0.5 McFarland. Wells of 6 mm in diameter were constructed and filled with varying quantities of AgNPs (1.5–0.375 mM). Plates were incubated at 37 °C for 24 h. The inhibitory zones were quantified in millimeters. Every experiment was conducted in triplicate to guarantee reproducibility [22].

The minimum inhibitory concentration (MIC) and minimum bactericidal concentration (MBC) of AgNPs were determined by the broth microdilution method in a sterile 96-well microtiter plates, as described previously [22, 23]. Serial dilutions of the AgNPs solution were prepared in Mueller-Hinton broth medium and inoculated with standardized bacterial suspensions. Plates were incubated at 37 °C for 24 h. The MIC was defined as the lowest concentration at which no visible bacterial growth was observed.

For determination of the MBC, aliquots from wells showing no visible growth were subcultured onto nutrient agar plates and incubated at 37 °C for 24 h. The MBC was defined as the lowest concentration resulting in no bacterial colony formation.

#### Time-dependent bacterial growth kinetics assay

The time-dependent bacterial growth kinetics of AgNPs against MRSA were evaluated at sub-inhibitory and inhibitory concentrations (0.5× MIC, MIC, and 2× MIC) to assess growth inhibition dynamics over time. Bacterial suspension was standardized to a 0.5 McFarland turbidity before exposure to the tested AgNPs. The cultures were incubated in a shaking incubator at 37 °C as previously described [22]. Samples were collected at pre-determined time intervals (0, 2, 4, 8, 12 and 24 h) and bacterial growth was determined by optical density (OD) measurement at 600 nm. Optical density measurements provide information on bacterial growth dynamics but are methodologically distinct from viable-count (CFU-based) assays, particularly for AgNPs [24].Untreated bacterial cultures served as growth controls, while solvent controls were included to rule out any effect of the dispersing medium.

#### Impact of AgNPs on the bacterial morphology by TEM

To assess the impact of AgNPs on the morphology and ultrastructural alterations of MRSA, bacterial cells were inoculated into nutrient broth and incubated at 37 °C for 24 h under shaking conditions. Untreated bacterial suspensions (control) and those subjected to the MIC of AgNPs for two hours were recovered via centrifugation and subsequently washed once with phosphate-buffered saline (PBS). The cell pellets were preserved in 2.5% glutaraldehyde overnight at 4 °C and then rinsed three times with 0.1 M phosphate buffer. Samples were dehydrated using a graded ethanol series, embedded in resin, and subsequently processed into ultrathin slices. Sections were subjected to staining with 3% uranyl acetate, followed by lead citrate [25]. Finally, the samples were examined by TEM (Hitachi H-7700, Japan) to observe structural changes induced by AgNPs treatment.

#### Confocal laser scanning microscopy (CLSM) analysis

The viability of bacteria after exposure to biosynthesized AgNPs was assessed by confocal laser scanning microscopy (CLSM) using the acridine orange/propidium iodide (AO/PI) dual fluorescent staining. MRSA isolates were cultured for bacterial attachment in sterile 8-well chamber slides at 37 °C for 24 h. Following incubation, non-adherent cells were removed by gentle washing with sterile PBS, and the attached bacterial cells were exposed to the biosynthesized AgNPs for two hours at 37 °C, while untreated wells served as controls.

After treatment, samples were washed twice with PBS and stained with AO/PI for 15 min in the dark. The stained bacterial cells were examined using a Leica TCS SP8 confocal microscope (Leica Microsystems, Germany) equipped with a Leica DMI8-CS inverted microscope stand and controlled by LAS X software (version 3.5.5.19976). AO fluorescence was visualized in the green channel, whereas PI fluorescence was detected in the red channel. Z-stack CLSM images were acquired and analyzed qualitatively for fluorescence distribution and quantitatively using Fiji software to assess bacterial viability following AgNPs exposure.

The bacterial viability index was calculated using the following equation:

$$\:Viability\:Index\:\%=\frac{Number\:of\:viable\:cells}{Number\:of\:viable\:cells\:+\:Number\:of\:dead\:cells}\:\times\:100$$

where AO-positive cells were considered viable, while PI-positive cells were considered membrane-compromised dead cells.

### In vivo study in a murine skin infection model

Thirty male albino rats, obtained from Cairo University, Egypt, with weights ranging from 180 to 230 g, were divided into three groups (*n* = 10). They were maintained under standard conditions, as previously designated [26]. An approval from the Faculty of Pharmacy Research Ethical Committee at Tanta University (TP/RE/5/26 p-002) was received. Group I served as the normal control, group II was the untreated group (0.9% normal saline), and group III included the NPs-treated group (125 µg/mL). After anesthesia, a small patch of hair was shaved from the backs of rats, followed by the formation of wounds [27]. All groups except for group I were inoculated with MRSA suspension (1 × 10⁶ CFU/mL) into each wound to establish infection, as previously described in comparable murine MRSA wound infection models [28, 29]. Each group was topically treated daily for two weeks [30].The assessment of wound healing was done over a two-week period, and the day of wound formation was considered day zero [27].

$$\:Wound\:healing\:\%=\frac{(Wound\:area\:at\:zero\:day-Wound\:area\:at\:the\:14th\:day)\:}{Wound\:area\:at\:zero\:day}\:\times\:100$$

On the fourteenth day, the skin tissues were homogenized and inoculated onto Muller-Hinton agar. Following overnight incubation of the plates at 37 °C, the CFU/g count was determined [30].

#### Histopathological and immunohistochemical inspection

Skin specimens were preserved in 10% neutral buffered formalin and embedded in paraffin wax. The paraffin-embedded sections underwent histological analysis utilizing hematoxylin and eosin (H&E) staining [31, 32]. Immunostaining for tumor necrosis factor-alpha (TNF-α), interleukin-6 (IL-6), and cyclooxygenase-2 (COX-2) was conducted utilizing monoclonal antibodies (Invitrogen, UK) [33].

### Statistics

Statistical analysis was conducted utilizing R software (R Foundation for Statistical Computing, Vienna, Austria). Data were presented as mean ± standard deviation (SD). Normality of all continuous variables was formally assessed using the Shapiro-Wilk test. Although a small number of groups showed significant departures from normality, one-way ANOVA was retained as the primary analysis because the study employed a balanced design with equal group sizes (*n* = 6 per group), under which ANOVA is generally considered robust to moderate deviations from normality. To further evaluate the robustness of the statistical conclusions, a non-parametric Kruskal-Wallis test was additionally performed as a sensitivity analysis for the inflammatory cytokine data, confirming significant overall differences among the experimental groups. A one-way analysis of variance (ANOVA) was employed to assess differences across the groups. Upon detecting significant differences, Tukey’s honest significant difference (HSD) post hoc test was utilized to conduct pairwise comparisons among groups. A *p*-value less than 0.05 was deemed statistically significant. Histological scores were evaluated using the Mann–Whitney U test because of the ordinal characteristics of the scoring method, the small sample size, and the restricted variability noted in some groups.

## Results

### Fungal isolate isolation and identification

Five different fungal isolates were obtained from the soil. Then, a preliminary screening was performed to select the most efficient fungal isolate for the biosynthesis of AgNPs. The antibacterial action of the biosynthesized AgNPs produced by each fungal isolate was then tested using the agar well diffusion. The fungal isolate that produces AgNPs showing the largest inhibition zone was selected for further AgNPs synthesis and characterization.

The screening results revealed variable antibacterial activities for the AgNPs synthesized by different fungal isolates, which indicates that the fungal source has a great influence on the bioactivity of the NPs. We noticed that AgNPs synthesized by *Fusarium sp.* exposed maximum antibacterial activity among all five tested isolates with regard to inhibition zones against the tested bacteria. Therefore, this isolate was selected as the most suitable fungal candidate for further molecular identification and AgNPs biosynthesis.

**Fig. 1:**
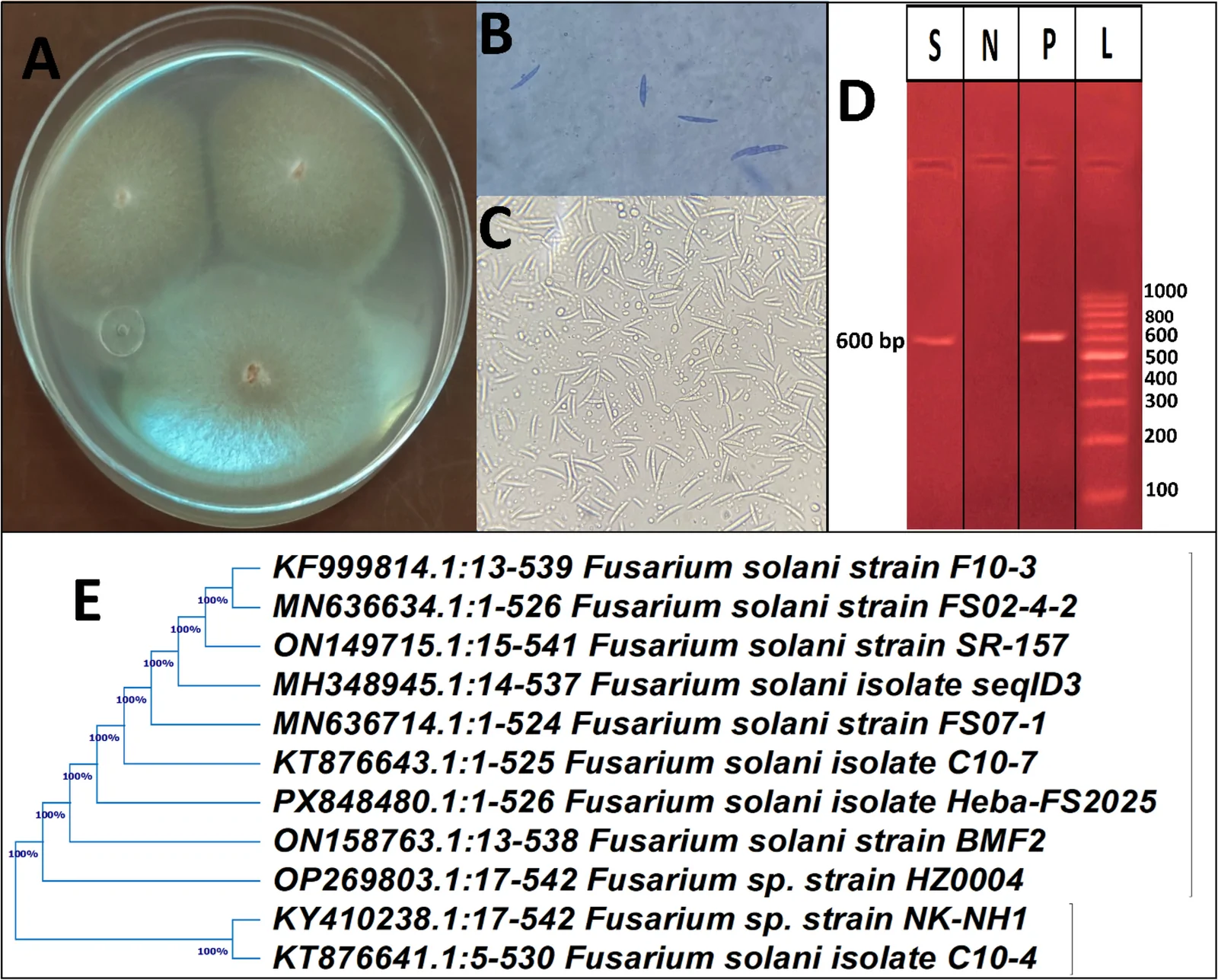
**A***Fusarium solani* colony on PDA, showing typical cottony/velvety white growth with pinkish/orange center. **B** Macroconidia under microscope, stained with lactophenol cotton blue, showing banana-shaped, multicellular structure with visible septa. **C** Macroconidia under microscope, unstained, showing natural transparent banana-shaped morphology and septation. **D** The amplification of the ITS region, producing a PCR product of approximately 600 bp. **E** phylogenetic tree of *Fusarium solani*

The *Fusarium* isolate was identified based on the macroscopic, microscopic, and molecular characteristics (Fig. 1). Colony growth on PDA medium was vigorous, forming white cottony mycelia which developed pinkish to violet pigmentation from the center with aging (Fig. 1A). Microscopically, the isolate displayed septate and branched hyphae, accompanied by curved (falcate) macroconidia and oval to elliptical microconidia (Fig. 1B and C), features typical of *Fusarium* species. The ITS rDNA sequencing was employed for the identification of the fungal isolate. The ITS region was successfully amplified, producing a PCR product with approximate size of 600 bp (Fig. 1D). The obtained sequence (526 bp) was subjected to BLAST analysis against the GenBank database and showed 100% identity and 100% query coverage with *Fusarium solani*. The E-value was 0.0, indicating a highly significant alignment and further confirming the identification of the isolate as *Fusarium solani*. The sequence was placed in GenBank under accession number PX848480, and the phylogenetic tree is exposed in Fig. 1E.

### GC–MS analysis of the ethyl acetate extract of *Fusarium solani*

It recognized a complex profile of secondary metabolites, ranging from volatile organic compounds to specialized bioactive molecules (Figure S1 and Table 1). The most abundant peak was for butane, 2,3-dimethyl- (43.05%), a branched alkane often associated with fungal volatile extraction processes. Beyond the primary hydrocarbons, the extract was characterized by a significant presence of nitrogen-containing heterocycles and phenolic derivatives.

**Table 1:** The GC-MS chemical profile of *Fusarium solani* extract

Peak	Retention time	Compound name	Area (%)	Chemical class
6	1.829	Butane, 2,3-dimethyl-	43.05	Alkanes (VOC)
7	1.886	1,3-Dihydro-5-phenyl-2 H-1,4-benzodiazepin-2-one	6.22	Benzodiazepine / Heterocycle
13	5.355	2-Hexanone, 3,3-dimethyl-	5.52	Aliphatic ketone
8	1.937	Hydrazine, 1,2-dimethyl-	5.10	Hydrazine derivative
23	42.810	N-Methyladrenaline (3TMS)	3.55	Catecholamine
24	43.105	4-Oxatricyclo[5.2.1.0(2,6)]dec-8-ene-3,5-dione	3.37	Cyclic anhydride
2	1.512	L-Alanine ethylamide, (S)-	2.18	Amino acid derivative
25	43.225	Ethyl homovanillate (TMS)	1.95	Phenolic ester
18	34.375	Oleic acid	1.94	Monounsaturated fatty acid
11	2.874	Methyl isobutyl ketone	1.33	Ketone
19	41.520	2,3-Dihydroxybenzoic acid (3TMS)	0.95	Phenolic acid

### Biosynthesis and characterization of AgNPs

A rapid color change from pale yellow to dark brown in the reaction mixture upon exposure to sunlight confirmed the reduction of Ag⁺ ions and the formation of AgNPs. No color change was observed in the control groups endorsing the essential role of both the fungal filtrate and sunlight in facilitation of the reaction.

**Fig. 2:**
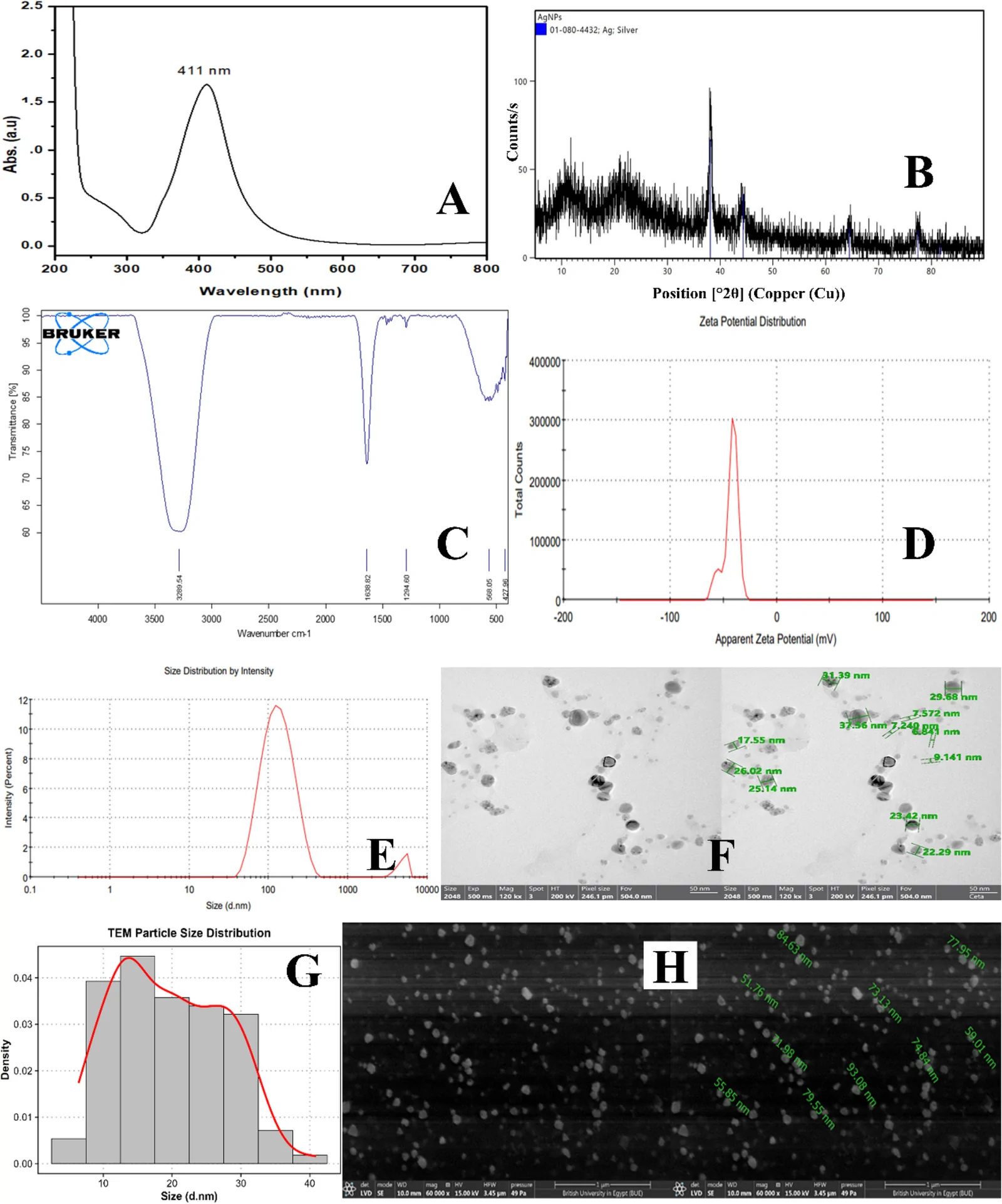
Comprehensive characterization of the biosynthesized AgNPs. **A** UV–Vis absorption spectrum confirming the formation of AgNPs. **B** X-ray diffraction (XRD) pattern indicating the crystalline structure with characteristic diffraction peaks. **C** Fourier-transform infrared (FTIR) spectra identifying surface functional groups, including amines and C–N bonds. **D** Zeta potential analysis demonstrating the surface charge and colloidal stability of the AgNPs. **E** Dynamic light scattering (DLS) analysis showing the Z-average hydrodynamic particle size and potential aggregation. **F** Transmission electron microscopy (TEM) micrographs revealing homogeneous, well-dispersed spherical AgNPs. **G** Particle size distribution histogram of the biosynthesized AgNPs derived from TEM measurements (*n* = 112), showing a mean particle diameter of 19.58 ± 7.90 nm. As the particle size data significantly deviated from a normal distribution (Shapiro–Wilk test, W = 0.9665, *p* = 0.0065), a kernel density curve (red) was superimposed to represent the observed size distribution. **H** Scanning electron microscopy (SEM) images showing the surface topology and morphology of the biosynthesized AgNPs

The UV–visible spectroscopy in Fig. 2A exposed a SPR peak at 410 nm, endorsing the reduction of Ag⁺ ions to AgNPs. The XRD pattern revealed the crystalline nature of the AgNPs by the sharp diffraction peaks (Fig. 2B). The FTIR spectra showed distinctive absorption bands matching to hydroxyl (–OH), carbonyl (C = O), and amine (–NH) groups, suggesting the existence of biomolecules associated with NP stabilization (Fig. 2C). The zeta potential value was recorded at -42.7 mV, demonstrating good colloidal stability and adequate surface charge for prevention of aggregation (Fig. 2D). The DLS analysis results of the AgNPs showed two peaks in the particle size analysis diagram, showing that the Z-average diameter of the biosynthesized AgNPs was 121.4 nm, with a PDI of 0.302, indicating a moderate size distribution (Fig. 2E). The TEM images demonstrated predominantly spherical NPs with an average diameter of 19.58 nm and a uniform distribution with minimal aggregation (Fig. 2F). Particle size distribution analysis of the TEM images (Fig. 2G), based on measurement of 112 individual particles using ImageJ, confirmed a predominantly narrow size range with a mean diameter of 19.58 ± 7.90 nm, closely corroborating the average diameter reported above. The size distribution significantly deviated from normality (Shapiro-Wilk test, *p* = 0.0065). The SEM micrographs (Fig. 2H) reveal the spherical shape of the AgNPs with particle sizes ranging from 30 to 90 nm with a relatively uniform distribution.

### Characterization of MRSA isolates

**Fig. 3:**
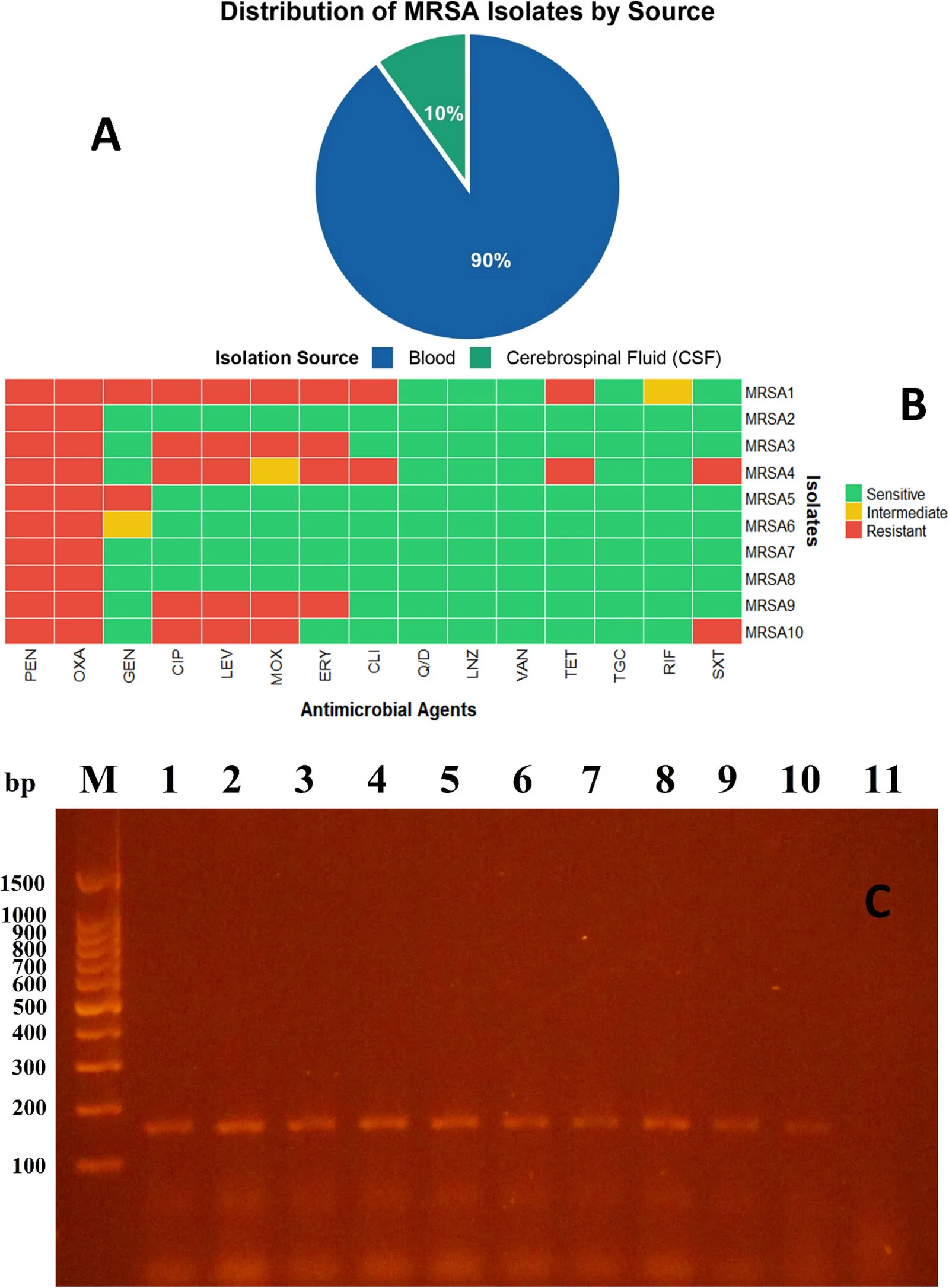
**A** Pie chart showing the clinical samples of the isolated MRSA isolates. **B** Heat map showing the antimicrobial susceptibility profile of the MRSA isolate. **C** Agarose gel electrophoresis of PCR amplification of the *mecA* gene in MRSA. Lane showing a distinct 163 bp band corresponding to the *mecA* amplicon; molecular size marker (100 bp DNA ladder); and negative control (no-template control)

MRSA isolates were isolated from nine blood samples and one cerebrospinal fluid (CSF) sample, obtained from ten distinct patients (Fig. 3A). MRSA isolates demonstrated a distinct antimicrobial susceptibility profile. They showed variable levels of resistance percentages toward the tested antibiotics Fig. 3B. Methicillin-resistant isolates phenotypically identified were verified by the revealing of the *mecA* gene. Agarose gel electrophoresis revealed a clear amplification product at 163 bp in the tested isolate, consistent with the expected size of the *mecA* gene. The positive control demonstrated successful amplification, while no bands were observed in the negative control, confirming assay specificity Fig. 3C. The demographic characteristics of the MRSA isolates, including patient age (range: 7–66 years) and sex, are detailed in Table S1, confirming that each isolate originated from a separate patient. Table S1.

### In vitro antibacterial activity of AgNPs against MRSA

AgNPs exhibited a dose-dependent antibacterial effect. Clear inhibition zones were observed with increasing concentrations, with the maximum zone of inhibition recorded at 32 mm for the highest concentration (1.5 mM) as shown in Table 2.

**Table 2:** Bacterial isolates and zone of inhibition of the AgNPs at different concentrations (0.375 mM, 0.75 mM, and 1.5 mM)

Isolate code	Inhibition Zone Diameter (mm) at		
	1.5 mM	0.75 mM	0.375 mM
MRSA1	20	16	11
MRSA2	30	28	20
MRSA3	30	26	20
MRSA4	19	15	10
MRSA5	20	16	12
MRSA6	20	18	10
MRSA7	24	22	19
MRSA8	32	30	28
MRSA9	18	16	12
MRSA10	19	15	11

The MIC values of the tested isolates ranged from 0.1875 to 0.375 mM, with MIC_50_ and MIC_90_ values of 0.1875 and 0.375 mM, respectively. The MBC values also ranged from 0.1875 to 0.375 mM, with MBC_50_ and MBC_90_ of 0.375 mM (Figure S2).

### Time-dependent bacterial growth kinetics assay

**Fig. 4:**
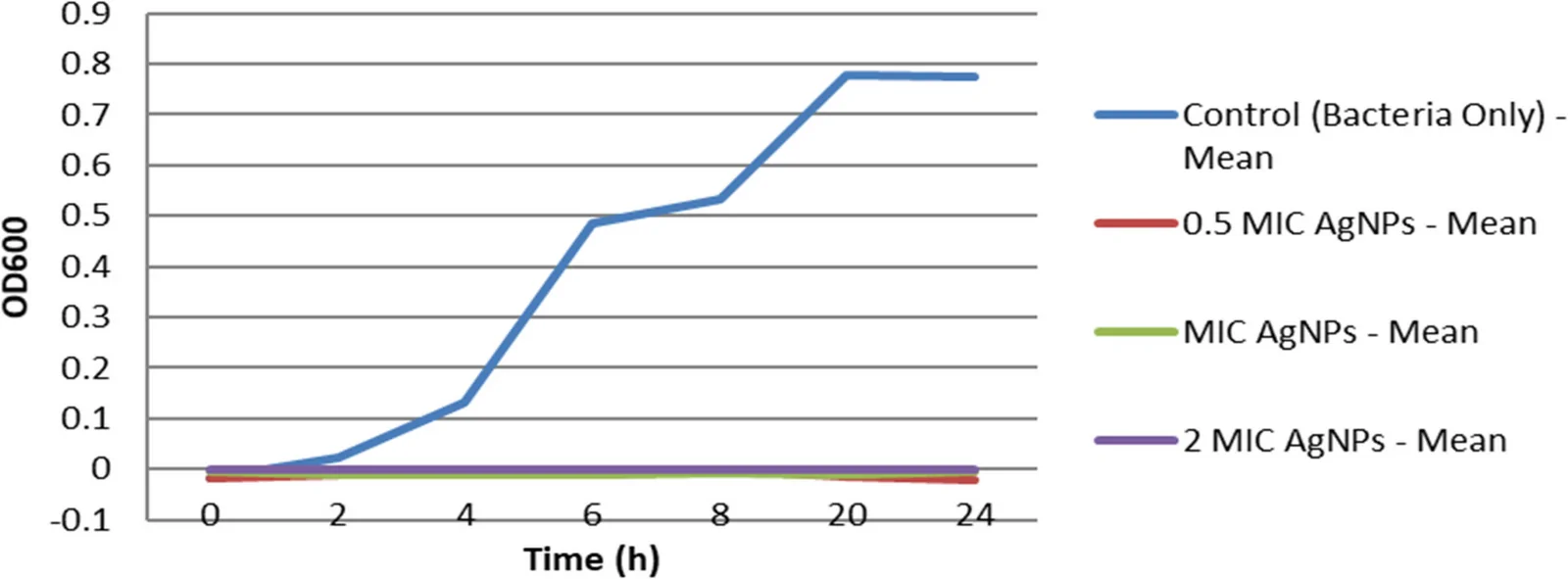
Time-dependent bacterial growth kinetics of the biosynthesized AgNPs on MRSA isolate at 0.5× MIC, 1× MIC, and 2× MIC

It showed that the AgNPs inhibited bacterial growth at 0.5× MIC, 1× MIC, and 2× MIC compared to the untreated bacterial cells, which showed evident growth over the incubation period. On the other side, no bacterial growth was perceived in the presence of AgNPs at all tested concentrations, with no clear concentration-dependent differences. OD₆₀₀ measurements indicated near-complete suppression of turbidity at all tested concentrations, including 0.5× MIC (Fig. 4).

### Morphological changes of the AgNPs-treated bacteria

**Fig. 5:**
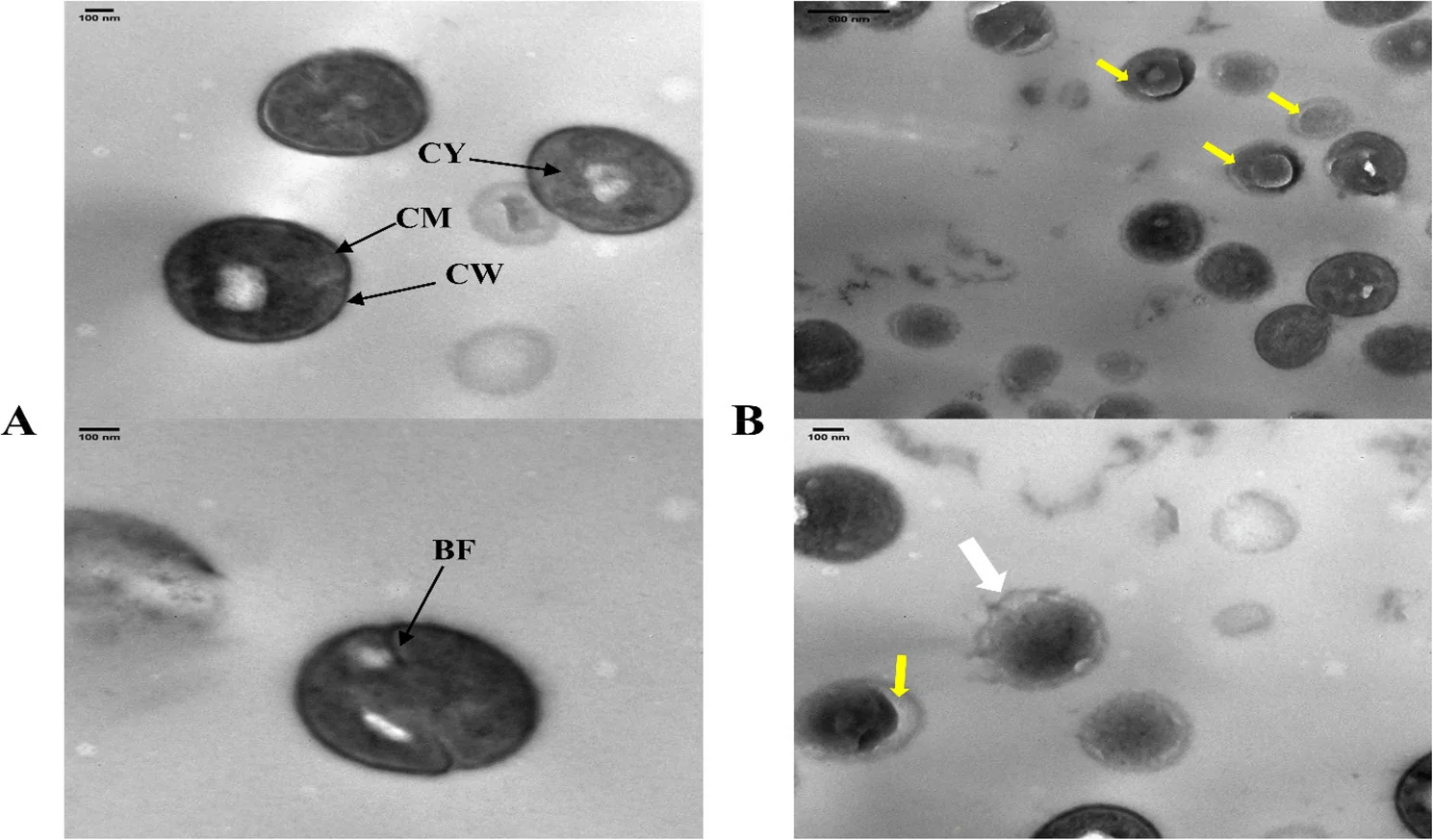
TEM images of bacteria showing: **A**) untreated bacterial cells undergoing binary fission (BF) with intact cell membrane (CM), cytoplasm (CY), and cell wall (CW), and **B**) AgNPs-treated bacterial cells displaying distorted cell wall (white arrow) and cell wall detachment from the plasma membrane (yellow arrow)

The TEM images of AgNPs-treated of MRSA isolate revealed morphological alterations, including distorted and detached cell walls from the plasma membrane, in contrast to the smooth and intact surfaces of untreated bacterial cells (Fig. 5).

### CLSM-based assessment of bacterial cell viability

**Fig. 6:**
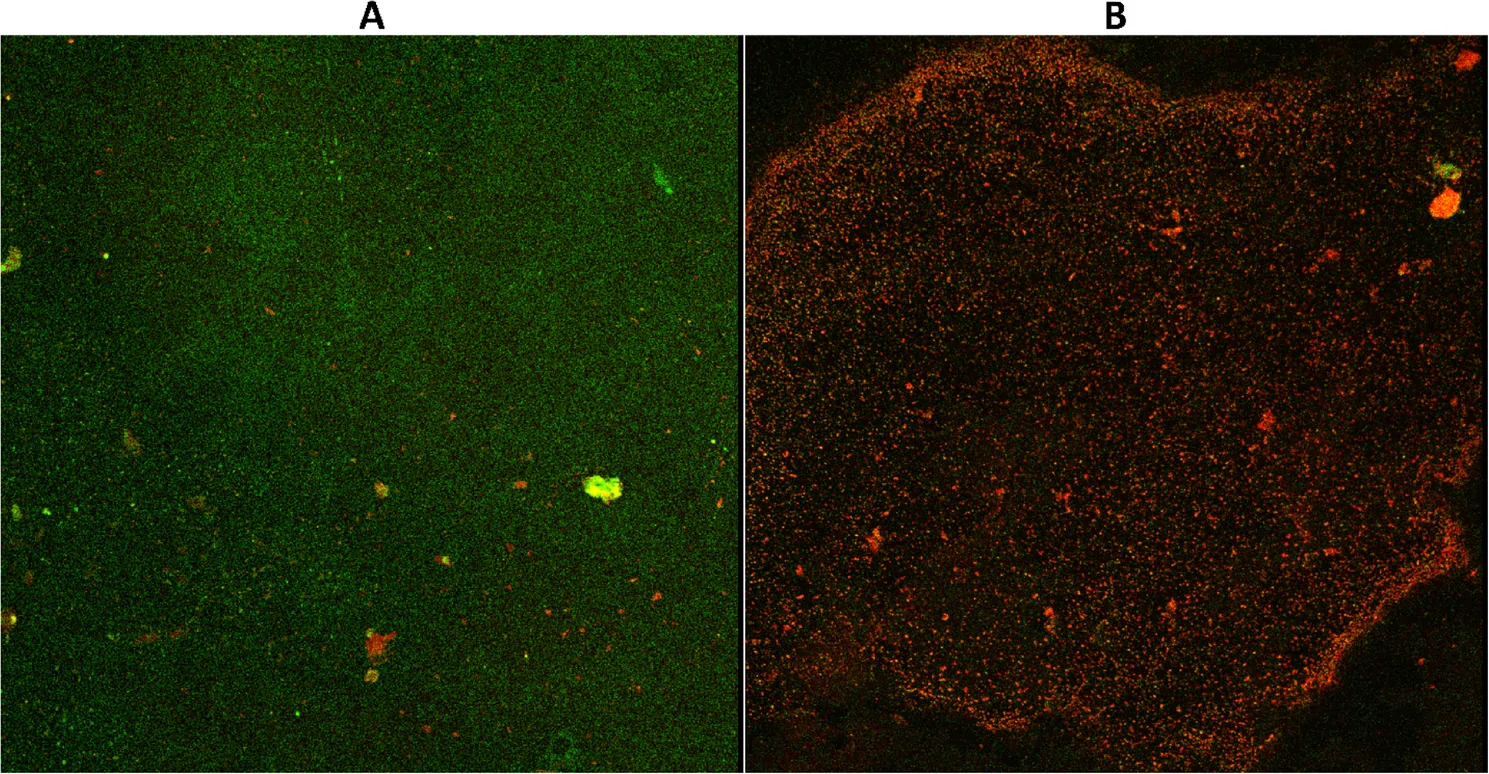
CLSM images of bacterial cells stained with AO/PI **A** Untreated bacterial cells showing predominant green fluorescence (AO), designating a great fraction of viable cells having undamaged membranes. **B** AgNPs-treated bacterial cells exhibiting amplified red fluorescence (PI) and reduced green signal (AO), indicating loss of membrane integrity and a noticeable decline in the viable bacterial cells

CLSM analysis with AO/PI dual fluorescent staining a clear difference in bacterial viability between untreated and AgNP-treated groups. The untreated MRSA cells showed mostly green fluorescence demonstrating a high fraction of viable bacteria with little red fluorescence indicating minimal membrane damage. In contrast, the treatment with biosynthesized AgNPs exhibited a noticeable rise in red fluorescence intensity with a marked decrease in green fluorescence intensity which indicates severe membrane damage and increased death of bacterial cells. Fiji (ImageJ 1.54p, NIH, USA) was employed for the quantitative fluorescence intensity analysis [34]. The viability index significantly decreased from 92.3% in the control group to 28.0% following AgNP treatment. This reduction was accompanied by a substantial increase in PI-positive (dead) cells and a corresponding decrease in AO-positive (viable) cells, demonstrating strong bactericidal activity of the biosynthesized nanoparticles. Overall, CLSM findings indicated that AgNPs exerted a pronounced antimicrobial effect against MRSA, primarily through membrane disruption leading to loss of bacterial viability (Fig. 6 and Table S2).

### In vivo antibacterial efficacy in a murine skin infection model

**Fig. 7:**
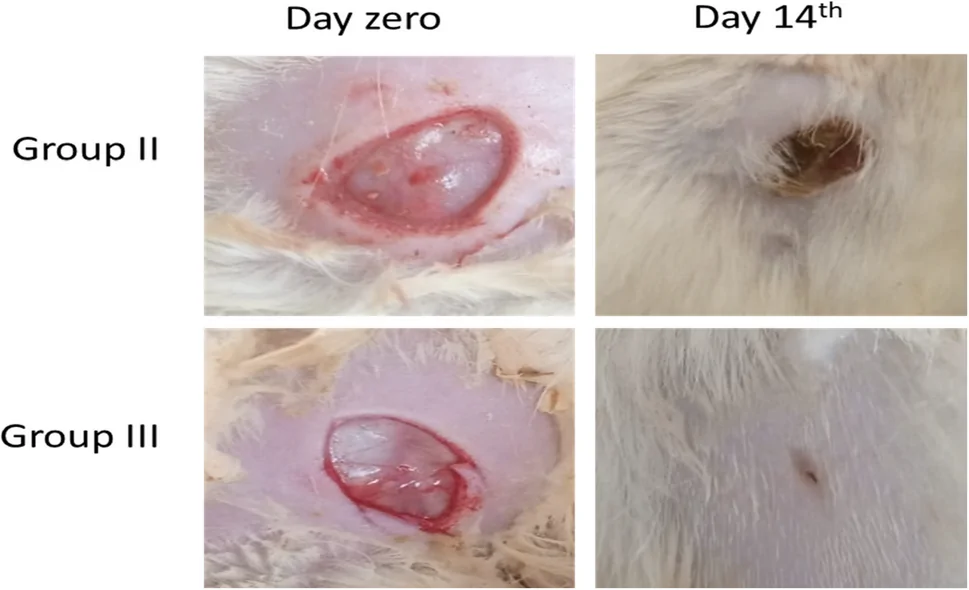
Macroscopic features of the wounds of groups II and III

The clinical features of the wounds were monitored from day 0 to day 14. Group III exhibited a noticeable healing compared with group II, with a considerable reduction in the wound size (Fig. 7).

Group III exposed an obvious wound healing on the 14th day with a substantial decline in the count of CFU/g (Table S3). These findings indicate that AgNPs treatment was associated with both substantial antibacterial activity and a pronounced enhancement in wound healing.

### Histopathology and immunohistochemistry

**Fig. 8:**
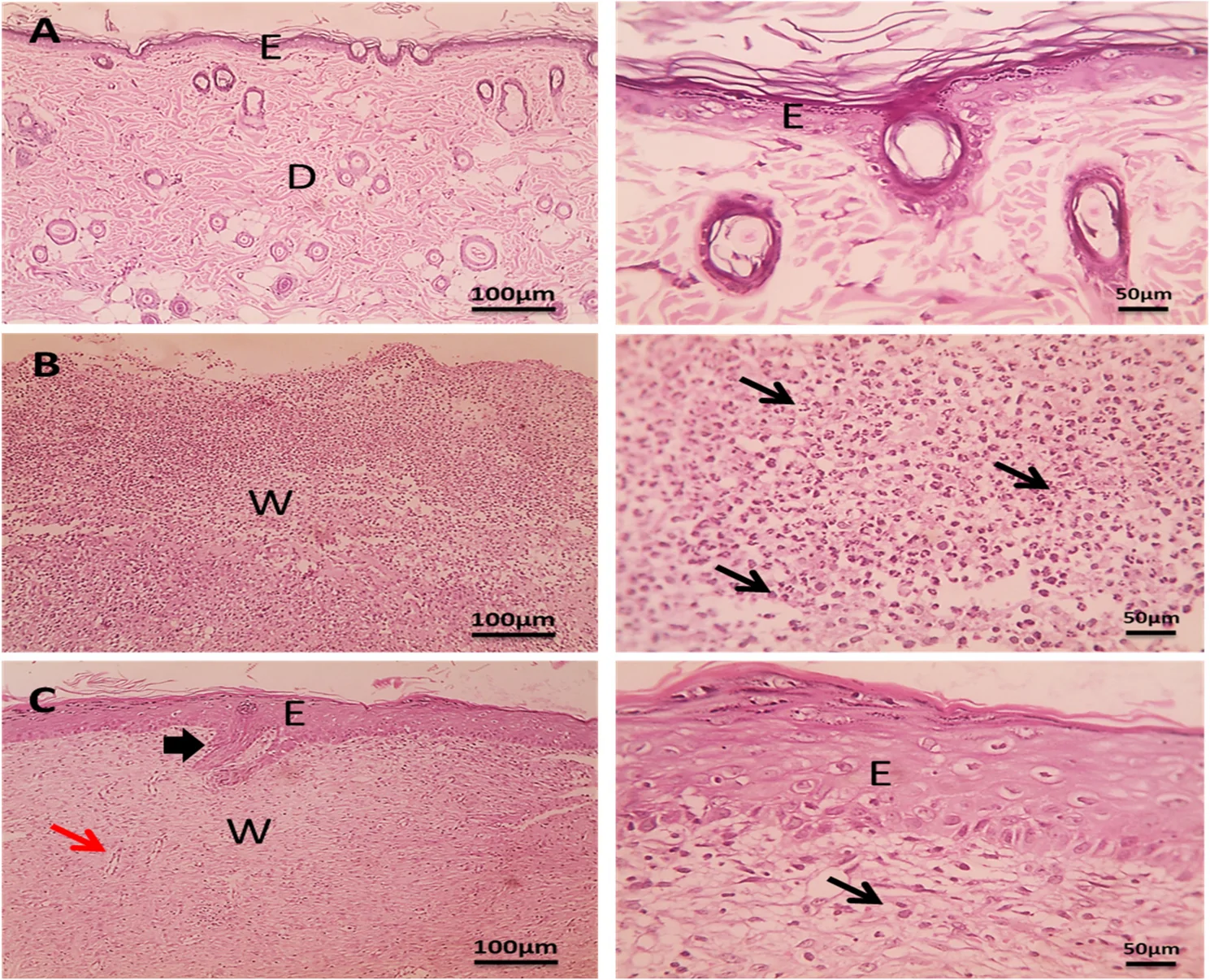
**A** Microscopic pictures of H&E skin section from group I displaying normal epidermis (E) and dermis (D) containing collagen fibers, mature fibroblasts, hair follicles, sebaceous glands, and fat cells. **B** Skin section from group II showing ulcerated skin and wound gap (W) filled with loose granulation tissue infiltrated by intense polymorphonuclear cells (black arrow). **C** Skin section from group III showing covered wound gap (W) with multilayered thick new epidermis (E) with rete pegs (thick arrow) and gap filled with organized granulation tissue, capillaries (red arrow), immature fibroblasts, and mononuclear cells (black arrow). ×100 bar 100 and ×400 bar 50

**Fig. 9:**
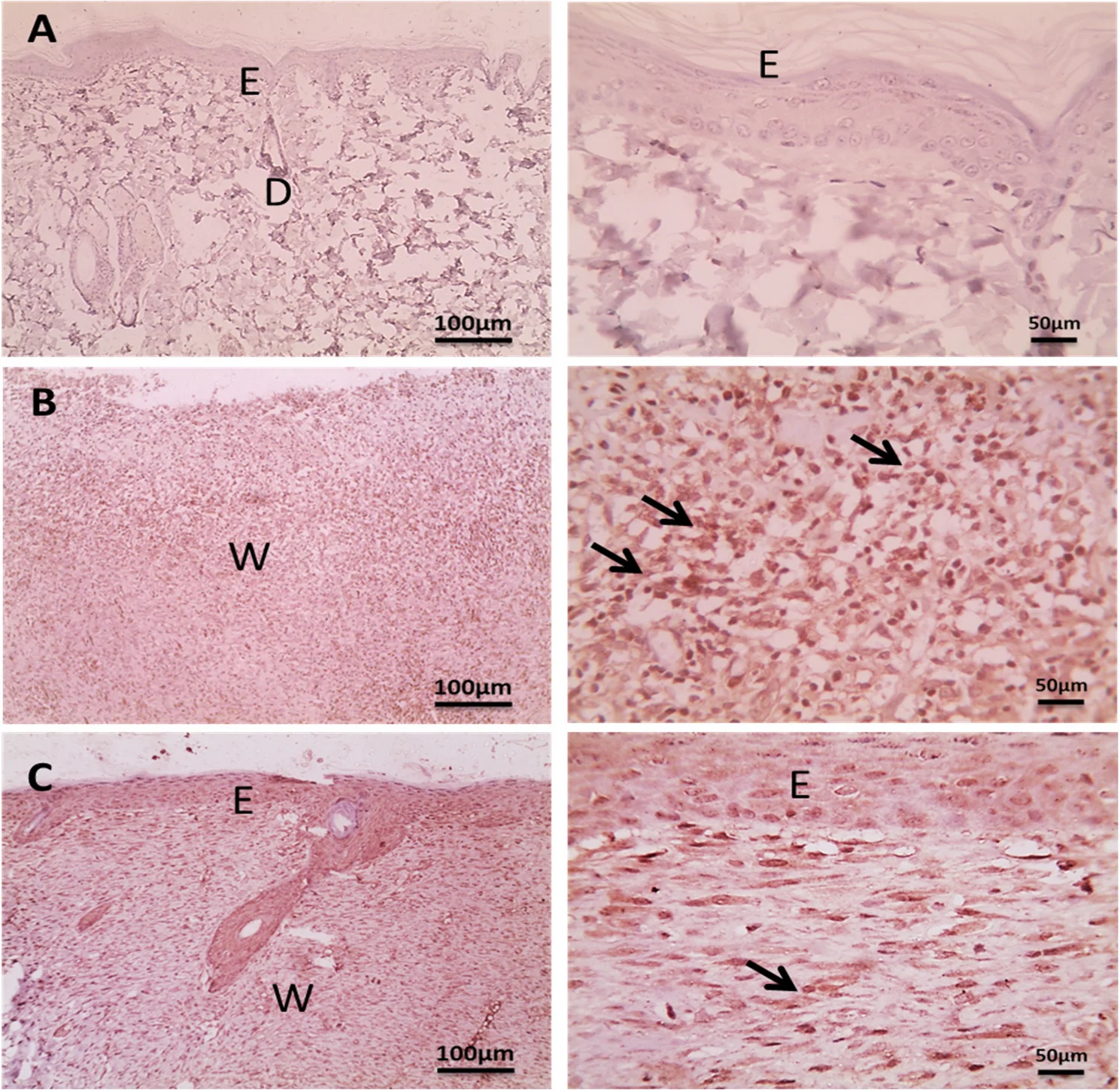
**A** Microscopic pictures of immunostained skin section from group I showing mild positive brown staining in dermis (D) (black arrow) against TNF-α. **B** Skin section from group II showing increased positive brown staining in wound gap (W) (black arrow) against TNF-α. **C** Skin section from group III showing decreased positive brown staining in wound gap (W) (black arrow) against TNF-α. ×100 bar 100 and ×400 bar 50

**Fig. 10:**
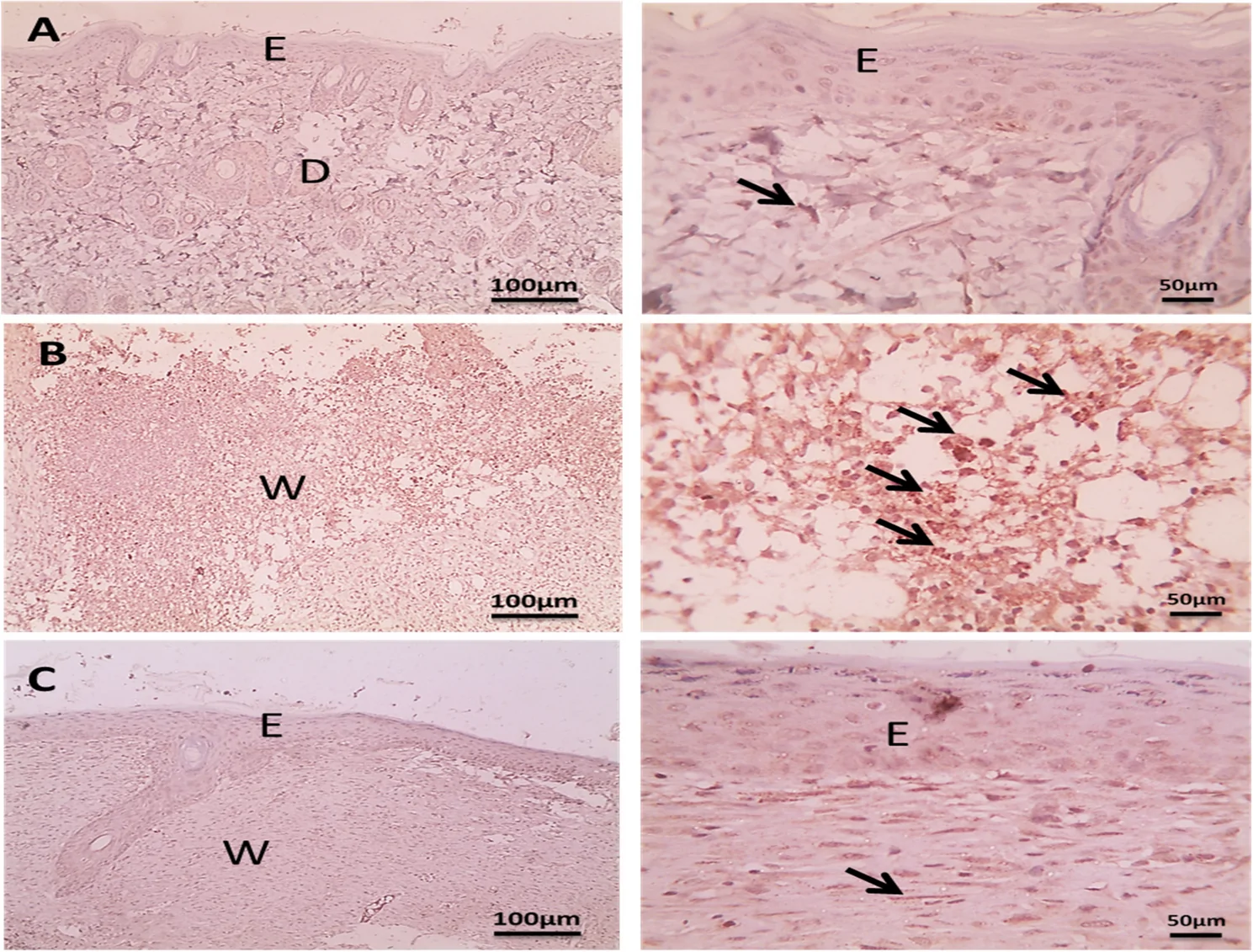
**A** Microscopic pictures of immunostained skin section from group I showing mild positive brown staining in dermis (D) (black arrow) against IL-6. **B** skin section from group II showing increased positive brown staining in wound gap (W) (black arrow) against IL-6. **C** Skin section from group III showing decreased positive brown staining in wound gap (W) (black arrow) against IL-6. ×100 bar 100 and ×400 bar 50

**Fig. 11:**
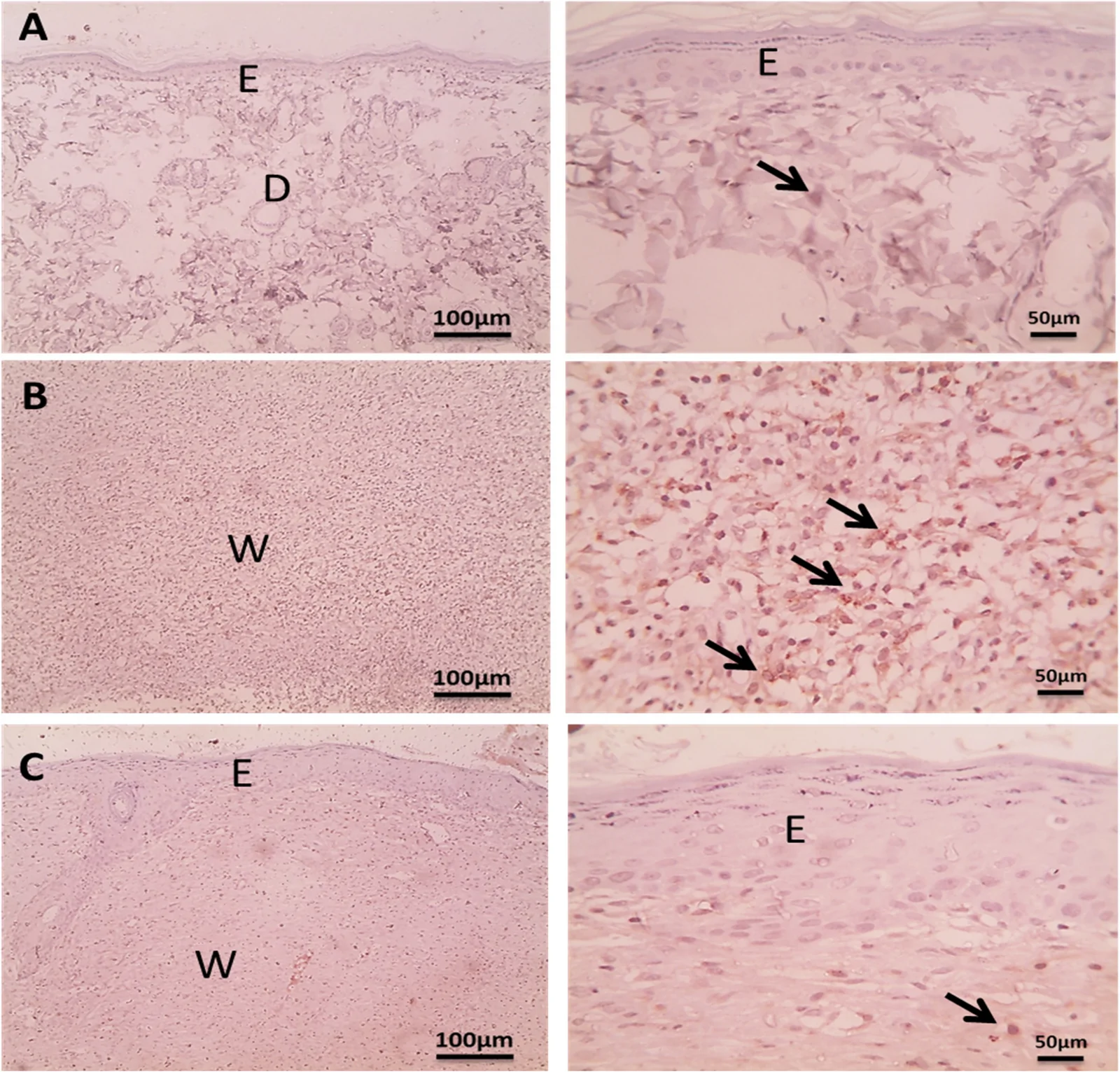
**A** Microscopic pictures of immunostained skin section from group I showing very mild positive brown staining in dermis (D) (black arrow) against COX-2. **B** Skin section from group II showing increased positive brown staining in wound gap (W) (black arrow) against COX-2. **C** Skin section from group III showing very mild positive brown staining in wound gap (W) (black arrow) against COX-2. ×100 bar 100 and ×400 bar 50

Figures 8, 9, 10 and 11 reveal the H&E-stained and immunostained skin sections.

**Fig. 12:**
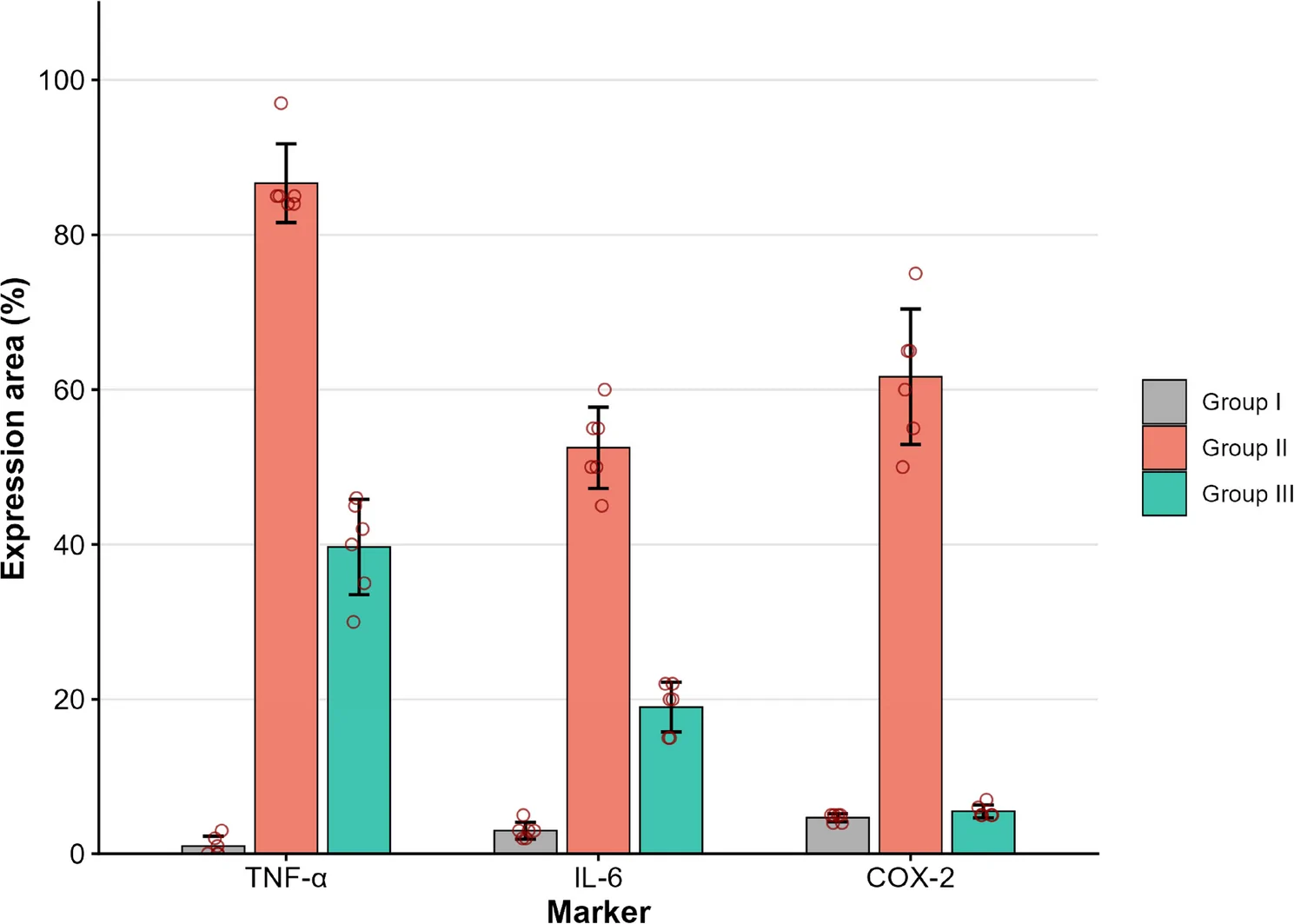
Immunohistochemical expression area (%) of inflammatory markers (TNF-α, IL-6, and COX-2) across the studied groups. Data are outlined as mean ± SD with individual replicate values shown as dots (*n* = 6 per group)

Figure 12 shows the immune cells area percentages of TNF-α, IL-6, and COX-2 in the experimental groups. Group II exposed a noteworthy rise in all inflammatory markers compared with group I. IL-6 increased from 3.00 ± 1.10% in the normal control group to 52.50 ± 5.24% in group II, while TNF-α significantly increased from 1.00 ± 1.26% to 86.67 ± 5.09%, and COX-2 significantly increased from 4.67 ± 0.52% to 61.67 ± 8.76%. After treatment with AgNPs (group III), expression of IL-6 and TNF-α were considerably decreased compared with group II, reaching 19.00 ± 3.22% and 39.67 ± 6.15%, respectively. COX-2 expression also decreased markedly in group III to 5.50 ± 0.84%, with no noteworthy difference compared with the normal control. Overall, AgNPs attenuated the inflammatory marker expression in a considerable manner in group II, with the most pronounced normalization observed for COX-2.

There were statistically significant differences in the expression levels of TNF-α, IL-6, and COX-2 among the experimental groups (*p* < 0.001 for all markers). For TNF-α, a noteworthy consequence of AgNPs was observed (F = 507, *p* = 1.69 × 10⁻14), with post hoc analysis indicating noteworthy differences between all pairwise comparisons. Similarly, IL-6 expression showed significant variation across groups (F = 294, *p* = 9.35 × 10⁻13), with Tukey’s test confirming significant differences between all groups (*p* < 0.05). COX-2 expression also demonstrated a significant group effect (F = 247, *p* = 3.27 × 10⁻12), with post hoc comparisons indicating statistically significant differences among all experimental groups. Overall, both treated groups exhibited reduced expression levels of inflammatory markers compared to the control group, with consistent patterns observed across all measured cytokines.

**Fig. 13:**
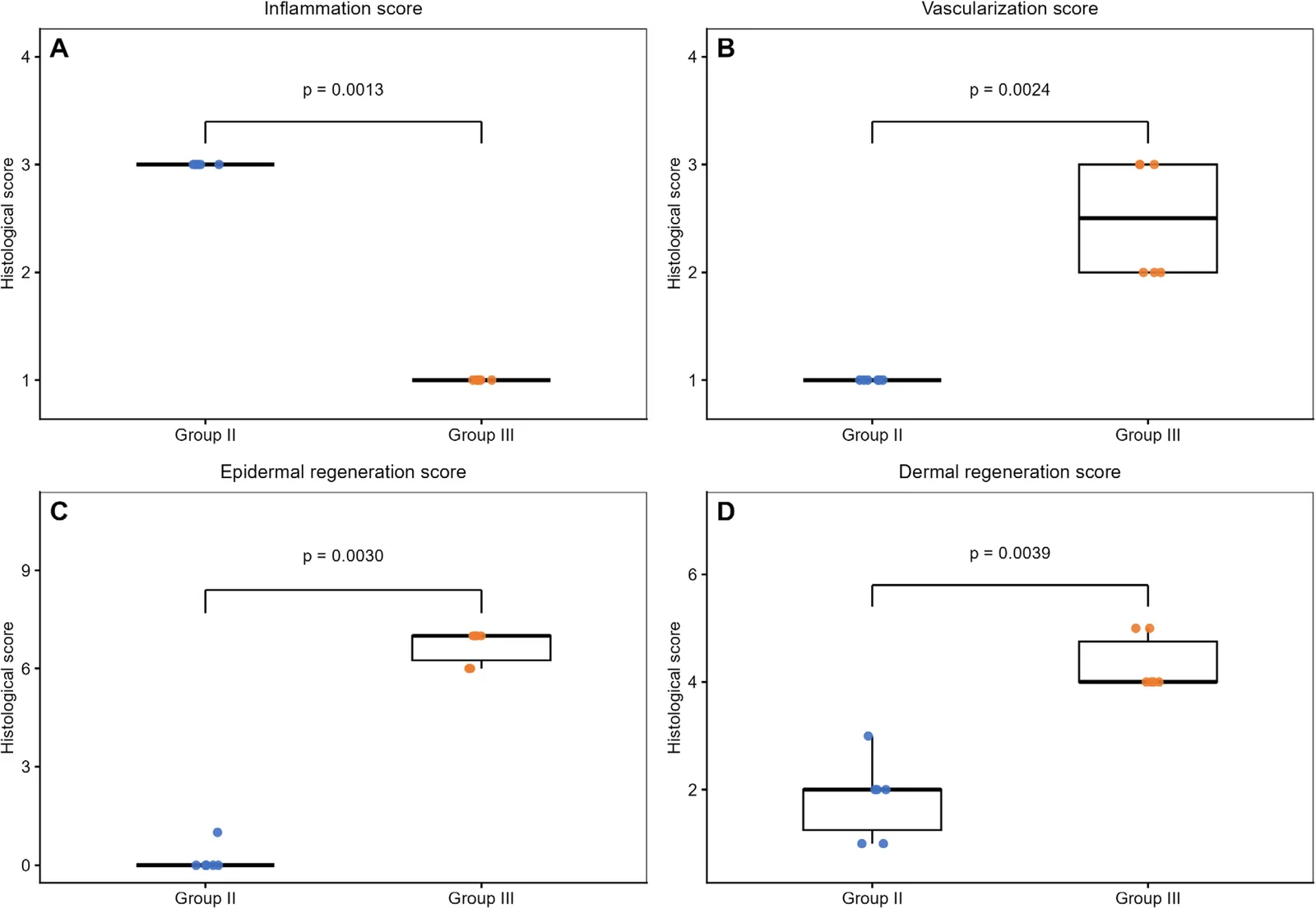
Histological scoring of H&E-stained tissues in the untreated group (Group II) and AgNPs-treated group (Group III). (**A**) Inflammation score, (**B**) Vascularization score, (**C**) Epidermal regeneration score, (**D**) Dermal regeneration score. Data are presented as individual data points with boxplots indicating the median and interquartile range

Compared with group II, the AgNPs-treated group showed significantly reduced inflammation, together with significantly increased vascularization, epidermal regeneration, and dermal regeneration, indicating a reduced inflammation and improved histological features of tissue repair and wound healing following AgNPs treatment (Fig. 13). Histological evaluation demonstrated significant differences between Group II and group III across all assessed parameters. Inflammation score was considerably deminished in group III compared with group II (*p* = 0.0013). In addition, vascularization score displayed a noteworthy increase in group III (*p* = 0.0024), indicating enhanced vascular response within the wound tissue. Epidermal regeneration score was significantly higher in group III compared with group II (*p* = 0.0030). Similarly, the dermal regeneration score demonstrated significant improvement in group III (*p* = 0.0039). Collectively, these findings indicate improved histological wound healing characteristics in group III relative to group II.

## Discussion

There is a great need to find novel sustainable alternatives to the traditional antimicrobials to treat different bacterial infections, especially MRSA infections. Here, we isolated *Fusarium solani* from the soil and employed this saprophytic fungus for AgNPs biosynthesis.

The GC-MS analysis of the *Fusarium solani* fungal extract illuminated various bioactive compounds contributing in AgNPs biosynthesis. The total ion chromatogram (TIC) showed several prominent peaks, the most abundant of which was a branched alkane. In addition to the primary hydrocarbons, the extract was rich in nitrogen-containing heterocycles and phenolic derivatives, which are important as reducing and capping agents. Among the metabolites identified by GC–MS, 2,3-dihydroxybenzoic acid is considered a plausible contributor to Ag⁺ reduction, as its catechol (ortho-dihydroxyphenyl) moiety is known to undergo oxidation to the corresponding quinone, thereby facilitating the reduction of Ag⁺ to Ag⁰ [35, 36]. Other phenolic constituents, including ethyl homovanillate and the putatively identified N-methyladrenaline, may also participate in Ag⁺ reduction through electron donation from their phenolic hydroxyl groups, although their individual contributions cannot be confirmed from the present data alone. In contrast, oleic acid is less likely to participate directly in silver ion reduction but may instead contribute to nanoparticle capping and colloidal stabilization, as commonly reported for fatty acids in fungal- and plant-mediated nanoparticle biosynthesis systems [37].

UV-visible spectroscopy gave stronger evidence of AgNPs formation. The colloidal suspension synthesized showed a clear SPR absorption peak at ~ 410 nm, which is in the usual range generally reported for AgNPs, designating the successful reduction of Ag+ ions to metallic Ag⁰ at the nanoscale [38]. The absorption bands corresponding to hydroxyl (–OH), carbonyl (C = O), and amine (–NH) groups in the FTIR analysis suggest the participation of fungal extracellular metabolites, particularly proteins, enzymes, and polysaccharides, in the reduction, capping, and stabilization of the AgNPs [39].

The XRD pattern, characterized by sharp diffraction peaks, confirmed the crystalline nature of the biosynthesized AgNPs, consistent with a face-centered cubic silver lattice commonly reported for fungal-mediated AgNPs [30, 31]. The well-defined crystallinity is particularly relevant because it indicates the successful formation of metallic silver and may contribute to the physicochemical stability and antimicrobial performance of the NPs, although silver ion release is also influenced by additional factors such as particle size, surface chemistry, and environmental conditions [40]. These findings suggest that the AgNPs synthesized in this study possess physicochemical characteristics favorable for consistent antimicrobial performance.

The TEM analysis showed that the biosynthesized AgNPs were mainly spherical, evenly dispersed, and less aggregated, with an average diameter of 19.58 nm. These structural characteristics align with a former study [41], and are comparable to the nanoscale diameter (19.13 ± 1.86 nm) recently reported for biologically synthesized AgNPs [42]. The small size of AgNPs at the nanoscale is biologically important because the smaller the particles, the larger the surface area, which may enhance their interaction with the surfaces of bacterial cells [43].

DLS exposed a Z-average hydrodynamic diameter of 121.4 nm and a PDI of 0.302. The hydrodynamic size was larger than the average particle diameter observed by TEM, but this difference can be expected since DLS measures the NPs in aqueous suspension and is representative of the hydrodynamic diameter, which takes into account the metallic core, surface-associated biomolecules, surrounding solvent layer, and any small particle clusters present in the colloid. This surrounding layer of adsorbed fungal proteins and metabolites effectively constitutes a biomolecular corona around the metallic core, analogous to the protein corona described for NPs in biological media, and is expected to substantially increase the apparent hydrodynamic diameter relative to the dry metallic core measured by TEM. On the other hand, TEM measures the dry metallic core of individual NPs. A similar discrepancy between DLS and TEM has been reported for mycogenic AgNPs [44]. The PDI value of 0.302 indicates a moderately polydispersed colloidal system. Although this value is close to the commonly accepted threshold of 0.3 for reasonably dispersed NPs suspensions, it also suggests that the biosynthesized AgNPs were not completely monodisperse. This aligns with a preceding report [45], which documented that PDI values should be interpreted carefully because DLS is sensitive to larger particles and aggregates. Thus, the DLS results support the formation of biologically capped AgNPs with acceptable colloidal dispersion, while also indicating some degree of size heterogeneity in suspension. These observations also have important implications for experimental reproducibility. The moderate polydispersity indicated by a PDI of 0.302 suggests that the synthesized AgNPs comprise a distribution of particle sizes rather than a completely monodisperse population. Because particle size and size distribution can influence physicochemical properties and biological interactions [45], maintaining a consistent particle size distribution is important for achieving reproducible biological performance. Therefore, minimizing size heterogeneity through careful control of synthesis parameters may further improve the reproducibility of future studies.

The zeta potential equal to − 42.7 mV designates a high negative surface charge on the biosynthesized AgNPs, and this suggests strong electrostatic repulsion between NPs. Since NPs with zeta potential values lower than − 30 mV are generally colloidally stable, the observed value supports the good dispersion stability of the biosynthesized AgNPs and indicates a reduced tendency toward aggregation, which aligns with earlier investigations [36, 37]. The negative surface charge perceived here may be assigned to ionized functional groups of fungal-derived capping biomolecules adsorbed onto the NP surface, such as proteins, amino acids, or other extracellular metabolites [46].

The particle size range observed by SEM (30–90 nm) was broader than the average diameter obtained by TEM (19.58 nm), likely reflecting differences in sample preparation and imaging resolution, and the greater likelihood of SEM images capturing surface-associated aggregates in dried samples, rather than exclusively individual dispersed NPs. Such discrepancies are commonly reported for biologically synthesized NPs, as each characterization technique measures a distinct physical property; relying on a single characterization technique may therefore lead to misleading conclusions, whereas integrating TEM, SEM, and DLS provides a more comprehensive and reliable assessment of nanoparticle morphology and colloidal behavior [45].

The antimicrobial susceptibility profile of the MRSA isolates revealed variable resistance patterns across the tested antibiotics, which is consistent with the recognized multidrug-resistant phenotype of clinical MRSA strains [47]. Confirmation of the *mec*A gene in all phenotypically resistant isolates further validates the molecular basis of methicillin resistance in this cohort. This observation aligns with reports indicating that SCCmec elements, particularly types II and III, frequently carry additional resistance determinants against other antibiotic classes alongside *mec*A, contributing to the broader multidrug-resistant phenotype commonly observed in clinical MRSA [48]. Consequently, evaluating AgNPs against molecularly confirmed MRSA provides a more clinically relevant assessment of their antimicrobial potential than testing phenotypically resistant isolates alone.

MRSA constitutes a major therapeutic challenge because its resistance limits empirical treatment options and often requires more targeted antimicrobial decision-making [47]. Herein, AgNPs demonstrated a clear concentration-dependent antibacterial effect on MRSA isolates using agar well diffusion. This agrees with an earlier work [49], which documented a positive relationship between AgNPs concentration and antibacterial efficacy against resistant *S. aureus* isolates. The results of agar well diffusion for AgNPs were interpreted with great caution due to their poor or uneven distribution through the agar. Certain factors, like particle size, aggregation, surface charge, and ion release, could influence results. Therefore, it was used as a preliminary screening test, and we detected the MIC and MBC values. The MIC values ranged from 0.1875 to 0.375 mM, while the corresponding MBC values fell within the same concentration range. This close MIC-MBC relationship suggests that AgNPs exerted a predominantly bactericidal effect under the experimental conditions used, particularly because the antimicrobial agents are commonly interpreted as bactericidal when the MBC is not more than fourfold higher than the MIC [50].

The time-dependent bacterial growth kinetics assay supported the sustained antibacterial potential of the tested AgNPs on MRSA. The AgNPs treated bacterial suspension at 0.5× MIC, 1× MIC, and 2× MIC showed markedly suppressed optical density growth curves relative to the untreated control, which showed progressive bacterial growth during incubation. It should be noted that optical density-based growth monitoring reflects overall culture turbidity rather than direct viable cell counts, and apparent complete growth suppression by this method does not necessarily equate to complete bacterial eradication; a similar pattern, in which OD-based kinetics curves indicated apparent complete growth suppression while colony-forming assays still detected viable bacteria, has been directly reported for AgNPs against MRSA [51]. This finding suggests that the AgNPs retained growth inhibitory activity over time within the tested concentration range, including the sub-MIC level, although confirmation via viable colony counts would be needed to establish whether this suppression reflects a bacteriostatic or bactericidal effect at this specific concentration.

TEM provided supportive ultrastructural evidence for the antibacterial effect of AgNPs on MRSA. In contrast to untreated cells, which showed relatively smooth and intact cell envelopes, AgNPs-treated cells exhibited clear morphological alterations, including cell wall deformation and apparent separation of the cell wall from the plasma membrane. This aligns with a former investigation showing AgNPs-associated cell envelope damage in *S. aureus*, including breakdown of the cell wall and alterations in bacterial morphology [52].

CLSM analysis gave supportive evidence of the consequences of AgNPs on the viability of the isolates. Untreated cells showed predominantly SYTO 9-associated green fluorescence and a high viability index of 92.3%, suggesting that most cells retained membrane integrity [53]. In contrast, exposure to AgNPs produced a marked shift toward reduced green fluorescence and increased PI-associated red fluorescence, with the viability index decreasing to 28%. The present CLSM findings are consistent with the TEM observations in MRSA isolates, suggesting that cell-envelope injury may contribute to the antibacterial effect of the AgNPs.

Despite these encouraging antibacterial and wound-healing findings, several challenges continue to hinder the clinical translation of AgNPs-based therapies. Systemic toxicity, bioaccumulation, and long-term safety following prolonged or repeated exposure remain incompletely characterized, particularly for green-synthesized formulations where batch-to-batch variability in physicochemical properties (e.g., size, PDI, surface chemistry) can substantially affect biological performance and reproducibility [54]. The absence of standardized synthesis, characterization, and dosing protocols across studies further complicates cross-study comparison and regulatory evaluation [54, 55]. Additionally, despite a growing body of preclinical evidence, clinical trials involving AgNPs-based formulations remain limited in number and scope, with regulatory frameworks for nanomaterial-based therapeutics still evolving [56]. Addressing these translational barriers through rigorous standardization, comprehensive toxicological profiling, and expanded clinical evaluation will be essential before fungal-derived AgNPs such as those described here can progress toward therapeutic application.

The in vivo wound infection model provided additional support for the antibacterial potential of the synthesized AgNPs. From day 0 to day 14, wounds in group III showed more evident healing progression than the untreated group, with a greater reduction in wound size. This finding suggests that AgNPs treatment was associated with improved wound contraction and tissue recovery in the infected wound environment. Similar findings have been reported in MRSA-infected skin wound models, where topical AgNPs-based treatments reduced wound size and improved healing progression compared with untreated infected controls [57, 58]. Likewise, a recent rat excision wound model infected with multidrug-resistant Pseudomonas aeruginosa demonstrated enhanced tissue regeneration following treatment with AgNP- and AuNP-based bionanoformulated creams [59]. The reduction in bacterial burden further supports an in vivo antibacterial effect of the AgNPs. Group III showed a substantial decline in CFU/g tissue compared with the untreated group, indicating a lower viable bacterial load at the wound site. This interpretation is consistent with recent MRSA and *S. aureus* wound infection studies in which AgNPs-based treatment reduced bacterial burden during wound healing [57, 60].

Histopathological assessment using H&E staining showed better re-epithelialization, reduced inflammatory-cell infiltration, and more organized granulation tissue in group III. This interpretation is supported by other studies [57, 58, 61].

Regarding the immunohistochemical assessment of TNF-α, IL-6, and COX-2 in the wound tissues, group III showed a reduced immunoreactivity of these inflammatory mediators compared with the untreated group, which aligns with a former investigation [61, 62]. It should be noted that the present study design cannot definitively distinguish whether this reduction reflects a direct immunomodulatory action of the AgNPs on host inflammatory signaling pathways, or an indirect consequence of reduced bacterial burden and the associated decline in bacterial-driven inflammatory stimulation, since both antibacterial and anti-inflammatory outcomes were assessed concurrently within the same treatment group. Both mechanisms are biologically plausible and are not mutually exclusive; AgNPs have been reported to modulate inflammatory signaling directly in some models, while in infection contexts, a substantial portion of cytokine reduction is also attributable to diminished bacterial load. Future studies incorporating a bacterial-clearance-matched comparator (e.g., an antibiotic-only treatment arm) would help delineate the relative contribution of each mechanism. Taken together, the in vivo findings indicate that AgNPs treatment was associated with improved wound-healing-related outcomes in the infected model, as reflected by reduced wound size, decreased CFU/g tissue, and improved histopathological and immunohistochemical findings. These results complement the in vitro antibacterial and ultrastructural findings and suggest that the AgNPs retained their antibacterial activity within the wound environment.

## Limitations

The physicochemical characterization was performed only in aqueous suspension; therefore, long-term colloidal stability under physiologically relevant conditions, including serum-containing media, physiological ionic strength, and varying pH, remains to be investigated. The exact pH of the synthesis reaction and the dry weight yield of the synthesized AgNPs were not recorded and should be systematically reported in future studies to enhance reproducibility and facilitate scale-up. Furthermore, this study focused on extracellular fungal filtrate-mediated biosynthesis, and a direct comparison with intracellular (lysate-mediated) synthesis was not performed. The time-kill assay relied on continuous optical density measurements without inclusion of a reference antibiotic control, which may partly explain the apparent growth suppression observed at sub-MIC concentrations. Also, the OD-based monitoring used in this study monitors culture turbidity rather than viable counts; upcoming studies should confirm bacteriostatic versus bactericidal activity at sub-MIC levels by colony counting. Future studies should incorporate a reference antimicrobial agent and finer sub-MIC concentration intervals to further validate antibacterial kinetics. Although membrane disruption and ultrastructural damage were confirmed by CLSM and TEM, the contribution of ROS to the antibacterial mechanism was not directly evaluated. Although the selected inoculum (1 × 10⁶ CFU/mL) successfully established a reproducible and persistent MRSA wound infection, future studies should evaluate the therapeutic efficacy of the formulation across a broader range of bacterial inoculum levels and infection severities. In addition, the observed reduction in inflammatory markers (TNF-α, IL-6, and COX-2) cannot be definitively attributed to a direct immunomodulatory effect of AgNPs, as no bacterial-clearance-matched comparator group was included. Additionally, silver ion release kinetics and in vivo biodistribution were not assessed, limiting mechanistic interpretation and safety evaluation. Future studies incorporating quantitative biodistribution analyses, such as ICP-MS, together with standardized toxicological assessment, will be important for supporting clinical translation. A further limitation of the present study is the absence of a standard antibiotic comparator group and interim (e.g., day 3, day 7) wound-healing assessments, both of which would provide a more comprehensive evaluation of relative treatment efficacy and healing kinetics; we recommend their inclusion in future studies.

## Conclusion

This study demonstrates the feasibility of using *Fusarium solani* as a sustainable biological platform for the production of AgNPs with promising antibacterial and wound-healing properties against MRSA-associated infections. The findings of this study highlight the potential of fungal-mediated nanotechnology as an alternative strategy for addressing antimicrobial resistance while supporting tissue repair. Nevertheless, further studies are required to elucidate the molecular mechanisms governing nanoparticle–host–pathogen interactions, establish standardized production protocols, evaluate long-term physicochemical stability and biosafety, and validate therapeutic efficacy in advanced preclinical models before clinical translation can be considered.

## Supplementary Information

Supplementary Material 1.

## Data Availability

No datasets were generated or analysed during the current study.
