# Supplementary material for: Eco-friendly biosynthesis of silver nanoparticles by saprophytic Fusarium solani as a sustainable therapeutic approach for the management of methicillin-resistant Staphylococcus aureus wound infections
Source: Microb Cell Fact. 2026 Oct 17;25:215. doi: 10.1186/s12934-026-03126-3 (PMC13675990; doi:10.1186/s12934-026-03126-3)
Supplement: Supplementary file 1 [file 12934_2026_3126_MOESM1_ESM.docx]

**Materials and methods**

#### **Sample collection and identification of bacterial isolates**

### The collection of samples and the identification of the bacterial isolates were done under strict aseptic conditions. All samples were immediately transported to the laboratory and processed by standard microbiological techniques to maintain viability and minimise contamination. Isolation of S. aureus was done using selective media (Mannitol Salt Agar (MSA)) for improved selective recovery and differentiation of the isolates. Colonies on MSA were yellow and presumptive for *S. aureus*. Further confirmation of the isolates was done by Gram staining, showing Gram-positive cocci in clusters and positive catalase and coagulase tests. Definitive identification was performed using the VITEK 2 automated identification system (bioMérieux, France) with a Gram-positive identification card, according to the manufacturer’s instructions.

**Antimicrobial susceptibility**

Methicillin resistance was initially determined phenotypically and then confirmed molecularly by PCR targeting the *mecA* gene encoding penicillin-binding protein 2a (PBP2a), the protein responsible for β-lactam resistance in S. aureus (Jonas et al., 2002; Snitser et al., 2020). PCR was carried out with specific primers (forward: 5′-ACTGCTATCCACCCTCAAAC-3′ and reverse: 5′-CTGGTGAAGTTGTAATCTGG-3′) with a predicted amplicon size of 163 bp. The reaction mixture (25 µL) contained 21 µL of Master Mix (Bioline, UK), 0.5 µL of each primer (10 pmol), and 3 µL of extracted DNA template. Thermal cycling conditions were: initial denaturation at 94 °C for 1 min, 35 cycles of denaturation at 94 °C for 1 min, annealing at 55 °C for 1 min, and extension at 72 °C for 1 min, followed by a final extension at 72 °C for 7 min. PCR products were separated using a 2.5% agarose gel stained with ethidium bromide and visualised under UV light. The 100 bp DNA ladder was used to detect the 163 bp band of *mecA* gene. For each PCR run, positive control (known *mecA*-positive *S. aureus* strain) and negative control (no-template control) were included to ensure accuracy and reliability. Confirmed isolates were kept in glycerol stocks at −80 °C and sub-cultured on nutrient agar prior to experimental use.

**Results**

**Figure S1**. Total ion chromatogram of *Fusarium solani* illustrating the distribution and abundance of identified secondary metabolites over a 45-minute run.

**Figure S2.** The MIC and MBC values for AgNPs against MRSA isolates.

**Table S1. Demographic characteristics and clinical sources of MRSA isolates.**

| **Isolate code** | **Clinical specimen** | **Sex** | **Age (years)** |
| --- | --- | --- | --- |
| **MRSA1** | Blood | male | 58 |
| **MRSA2** | Blood | male | 27 |
| **MRSA3** | Blood | female | 66 |
| **MRSA4** | Blood | female | 52 |
| **MRSA5** | Blood | male | 34 |
| **MRSA6** | CSF | male | 44 |
| **MRSA7** | Blood | female | 60 |
| **MRSA8** | Blood | female | 7 |
| **MRSA9** | Blood | female | 66 |
| **MRSA10** | Blood | male | 39 |

**Table S2. CLSM-based quantitative analysis of biofilm viability and morphology in control and AgNPs-treated groups.**

| Group | **Live cells** | Dead cells | Live/Dead ratio | Dead cells (%) | Viability index (%) |
| --- | --- | --- | --- | --- | --- |
| Control (Bacteria Only) | 60367 | 5049 | 11.96 | 7.7 | 92.3 |
| Treated (MIC, 2 hr) | 20161 | 51723 | 0.39 | 72 | 28.0 |

**Table S3.** Effect of AgNPs treatment on the bacterial load and wound healing percentage

| **Parameter** | **Positive control group (mean ± SD)** | **AgNPs-treated group (mean ± SD)** |
| --- | --- | --- |
| Log CFU/g | 9.90 ± 0.26 | 1.10 ± 0.10 |
| Wound healing percentage (%) | 12.33 ± 2.08 | 95.33 ± 2.08 |
